# Diagnostic value of neutrophil gelatinase-associated lipocalin, cystatin C, and soluble triggering receptor expressed on myeloid cells-1 in critically ill patients with sepsis-associated acute kidney injury

**DOI:** 10.1186/s13054-015-0941-6

**Published:** 2015-05-06

**Authors:** Xingui Dai, Zhenhua Zeng, Chunlai Fu, Sheng’an Zhang, Yeping Cai, Zhongqing Chen

**Affiliations:** Department of Critical Care Medicine, Nanfang Hospital, Southern Medical University, 1838 Guangzhou Avenue North, Guangzhou, Guangdong 510515 China; Department of Critical Care Medicine, the First People’s Hospital of Chenzhou, Luo Jia Jin Street 108, Chenzhou, Hunan 423000 China; Guangdong Key Laboratory of Shock and Microcirculation Research, Department of Pathophysiology, Southern Medical University, 1838 Guangzhou Avenue North, Guangzhou, 510515 China

## Abstract

**Introduction:**

Neutrophil gelatinase-associated lipocalin (NGAL), cystatin C (Cys-C), and soluble triggering receptor expressed on myeloid cells-1 (sTREM-1) are novel diagnostic biomarkers of acute kidney injury (AKI). We aimed to determine the diagnostic properties of these biomarkers for detecting AKI in critically ill patients with sepsis.

**Methods:**

We divided 112 patients with sepsis into non-AKI sepsis (n = 57) and AKI sepsis (n = 55) groups. Plasma and urine specimens were collected on admission and every 24 hours until 72 hours and tested for NGAL, Cys-C, and TREM-1 concentrations. Their levels were compared on admission, at diagnosis, and 24 hours before diagnosis.

**Results:**

Both plasma and urine NGAL, Cys-C, and sTREM-1 were significantly associated with AKI development in patients with sepsis, even after adjustment for confounders by using generalized estimating equations. Compared with the non-AKI sepsis group, the sepsis AKI group exhibited markedly higher levels of these biomarkers at diagnosis and 24 hours before AKI diagnosis (*P* <0.01). The diagnostic and predictive values of plasma and urine NGAL were good, and those of plasma and urine Cys-C and sTREM-1 were fair.

**Conclusion:**

Plasma and urine NGAL, Cys-C, and sTREM-1 can be used as diagnostic and predictive biomarkers for AKI in critically ill patients with sepsis.

## Introduction

Sepsis is well known as a life-threatening syndrome that develops as a result of systemic inflammatory response to infection; it remains the leading cause of death and has a 30% to 40% mortality rate in the intensive care unit (ICU) [1,2]. Acute kidney injury (AKI) is one of the leading causes of sepsis-related death in critically ill patients, and 50% of all cases of AKI are considered to be associated with sepsis [3,4]. The exact pathogenesis and clinical characteristics leading to AKI in patients with sepsis remain elusive, and diagnostic tools that can detect AKI at an early stage are lacking, and this may account for the very high morbidity and mortality rates of sepsis-associated AKI. Currently, the diagnosis of AKI is based mainly on an increase in the serum creatinine (SCr) level, which indicates loss of excretory renal function according to the Risk, Injury, Failure, Loss, and End-stage Kidney disease (RIFLE) [5], Acute Kidney Injury Network (AKIN) [6], and Kidney Disease: Improving Global Outcomes (KDIGO) criteria [7]. However, the SCr level does not accurately reflect the glomerular filtration rate (GFR) in patients with sepsis, as GFR is regulated by tubular creatinine secretion and non-renal factors such as liver function, muscle mass, and non-renal gastrointestinal elimination [8]. SCr is also recognized as a late marker of kidney injury [9,10]. For these reasons, it is vital to identify other indicators that can be used for early diagnosis of sepsis-associated AKI.

Numerous potential markers for the early diagnosis of AKI have been under study in the last decade. Among these biomarkers, neutrophil gelatinase-associated lipocalin (NGAL), cystatin C (Cys-C), and soluble triggering receptor expressed on myeloid cells-1 (sTREM-1) have received the most attention. Although several studies have already focused on the performance of these biomarkers for diagnosing AKI in patients with or without sepsis [11-18], the diagnostic properties of these biomarkers remain a matter of debate because of the complexity of clinical conditions and pathological processes. NGAL, a 25-kDa protein that covalently binds to gelatinase from neutrophils, is rapidly released by activated neutrophils in response to ischemic or toxic damage [11,19]. According to experimental and clinical studies, NGAL is one of the most promising early biomarkers of AKI [11,18]. Cys-C, another functional biomarker, has been found to be superior to SCr as a marker of renal function [20]. However, its diagnostic value is not clear. Most research demonstrates that Cys-C functions well as a predictor of AKI [12,14,21], but a few studies have shown that it is a poor predictor [15,22]. The expression of TREM, a glycoprotein of the immunoglobulin superfamily, in neutrophils and monocytes is upregulated in the presence of infection [23,24]. Its role is to amplify the innate inflammatory response and sepsis mediated by the engagement of Toll-like receptors and nucleotide-binding oligomerization domain (NOD)-like receptors [25-27]. sTREM-1, the soluble form of TREM-1, is extensively released into peripheral circulation upon upregulation of the expression of TREM-1 [25,26]. Su *et al*. [16] have reported that this 27-kDa protein can be excreted by the kidney provided that kidney injury exists. An increasing number of studies indicate that patients with sepsis have increased sTREM-1 levels in body fluid samples, which are closely related to the severity of infection and are predictive markers of prognosis [28].

Despite such extensive research into these markers, the diagnostic properties of NGAL, Cys-C, and sTREM-1 with regard to AKI occurrence in patients with sepsis need to be clarified. This study was designed to determine the diagnostic and predictive value of these biomarkers for sepsis-associated AKI in a general ICU population.

## Methods

### Study population

This prospective observational study was conducted at the general ICU of the First Peoples’ Hospital of Chenzhou, Hunan Province, China. The protocol was approved by the Ethics Committee (project 2012033-003) of the First Peoples’ Hospital of Chenzhou. Patients or their family members were fully informed of the study details and signed the informed consent forms of their own accord. All of the consecutive eligible patients were selected from among inpatients who were hospitalized between March 2012 and March 2014. One hundred twelve patients with sepsis were included in the study and were divided into two groups: a non-AKI sepsis group (n = 57) and an AKI sepsis group (n = 55). The AKI sepsis group comprised sepsis patients who developed AKI during the first week. The patients were screened daily for AKI occurrence for up to days 7.

### Inclusion and exclusion criteria

Consecutive adult (at least 18 years old) sepsis patients admitted to the ICU were assessed for inclusion. The following patients were excluded: (1) those who did not give their consent or who declined treatment during the period of observation; (2) those who were exposed to the presence of radiocontrast agents or nephrotoxin drugs 5 days prior to admission; (3) those with pre-existing AKI (known in any stage of AKI prior to admission); (4) those with chronic kidney disease (CKD), defined according to the definition of the National Kidney Foundation as kidney damage or GFR of less than 60 mL/min per 1.73 m^2^ for at least 3 months, irrespective of the cause [29]; (5) those who had undergone renal transplant; (6) those who required renal replacement therapy (RRT); (7) those with anuria; (8) those with cancer; (9) those who had participated in other studies; (10) those who had contracted AIDS; and (11) those who had undergone high-dose steroid treatment.

### Definitions

According to the diagnostic criteria of the 2001 International Sepsis Definition Conference [30], sepsis is a systemic, deleterious host response to infection leading to systemic inflammatory response syndrome, which is characterized by two or more of the following conditions: hypothermia or fever (body temperature of less than 36°C or more than 38.5°C, respectively), tachycardia (>90 beats per minute), tachypnea (>20 breaths per minute or partial pressure of arterial carbon dioxide (PaCO_2_) of less than 32 mm Hg during mechanical ventilation), leukocytosis (>12,000/mm^3^), leukopenia (<4,000/mm^3^), and an increase in the number of immature band forms (>10%).

According to the 2012 KDIGO criteria [7], which are based on the RIFLE/AKIN definitions, we used the urine output and SCr components as indicates of AKI. The AKI is characterized by a 48-hour absolute increase in SCr of at least 26.4μmol/L, and an increase of at least 50% from baseline that is known or presumed to have occurred within the prior 7 days, and a decline in urine output to not more than 0.5mL/kg per hour for at least 6 hours.

### Data collection

When the patients were admitted to the ICU, data on the baseline characteristics, including age, gender, etiological factors, and underlying diseases, were collected. SCr levels were obtained on admission and every 12 hours (9 a.m. and p.m. ± 1 hour), and urine output was recorded every hour for diagnosing AKI. To determine the severity of inflammation, the white blood cell (WBC) count and the level of C-reactive protein (CRP) and procalcitonin (PCT) were determined. Other physiological and clinical information was collected and scored by using the Sequential Organ Failure Assessment (SOFA) score and Acute Physiology and Chronic Health Evaluation II (APACHE II) score.

### Sample processing and measurement

Blood and urine samples were obtained on admission and every 24 hours up to 72 hours for measuring the NGAL, Cys-C, and sTREM-1 levels. Blood was centrifuged at 3,000 revolutions per minute (rpm) for 15 minutes, and urine was centrifuged at 2,000 rpm for 5 minutes. The supernatants were transferred to Eppendorf tubes and stored at −80°C. All of the specimens were renumbered before the experiment. The plasma NGAL level was determined by using a Triage NGAL Assay (Alere Inc., San Diego, CA, USA), and the measurable range was 15 to 1,300 ng/mL. The urine NGAL level was analyzed by using a NORMAN-2 scattering turbidimetry analyzer with an NGAL Assay (Norman Inc., Nanjing, China), and the measurable range was 0 to 4,000 ng/mL. The Cys-C level was measured by using an automated chemistry analyzer (Hitachi 7600 Clinical Analyzer; Hitachi, Tokyo, Japan) with a latex immunoturbidimetry assay. The level of sTREM-1 was determined by using a double-antibody sandwich enzyme-linked immunosorbent assay (ELISA) (R&D Systems Inc., Minneapolis, MN, USA) with a measurable range of 0 to 4,000 pg/mL. ELISA was performed in duplicate, and other assays were performed in strict accordance with the instructions of the manufacturers. Laboratory investigators were blinded to the clinical information throughout the study.

### Statistical analysis

Results for continuous variables with normal distribution, including age, mean arterial pressure (MAP), APACHE II and SOFA scores, and SCr, are presented as mean ± standard deviation. The Student’s *t* test was used to compare means between the two groups. Results for continuous variables that were not normally distributed, including WBC counts, CRP, PCT, urine NGAL, plasma NGAL, plasma sTREM-1, urine sTREM-1, plasma Cys-C, and urine Cys-C, are presented as the median values (25th and 75th percentiles) and were compared by using the Mann-Whitney *U* test. Results for qualitative variables were expressed as number (percentage) and compared between groups by using the chi-square test or Fisher’s exact test. Survival rates were calculated by using the Kaplan-Meier method, and between-group differences were assessed by using the log-rank test. Odds ratios and the corresponding confidence intervals (CIs) for models of these biomarkers in plasma and urine with the risk of AKI occurrence in sepsis were analyzed by using generalized estimating equations (GEEs). The variables were combined to create three models. In model 1, no moderator variables were adjusted; in model 2, SCr was used as the moderator variable; and in model 3, MAP, APACHE II scores, SOFA scores, and PCT were adjusted in addition to SCr. Receiver operating characteristic (ROC) analysis was used to explore the ability of these biomarkers to predict AKI occurrence in patients with sepsis at diagnosis and 24 hours before diagnosis. Areas under the receiver operating characteristic curves (AUROCs) were used to evaluate how well the model could distinguish AKI patients with sepsis from non-AKI patients with sepsis. Statistical analyses were conducted by IBM SPSS 19.0 (SPSS, Chicago, IL, USA), and a two-tailed *P* value of less than 0.05 was considered to indicate statistical significance.

## Results

### Patient characteristics

**Figure 1:**
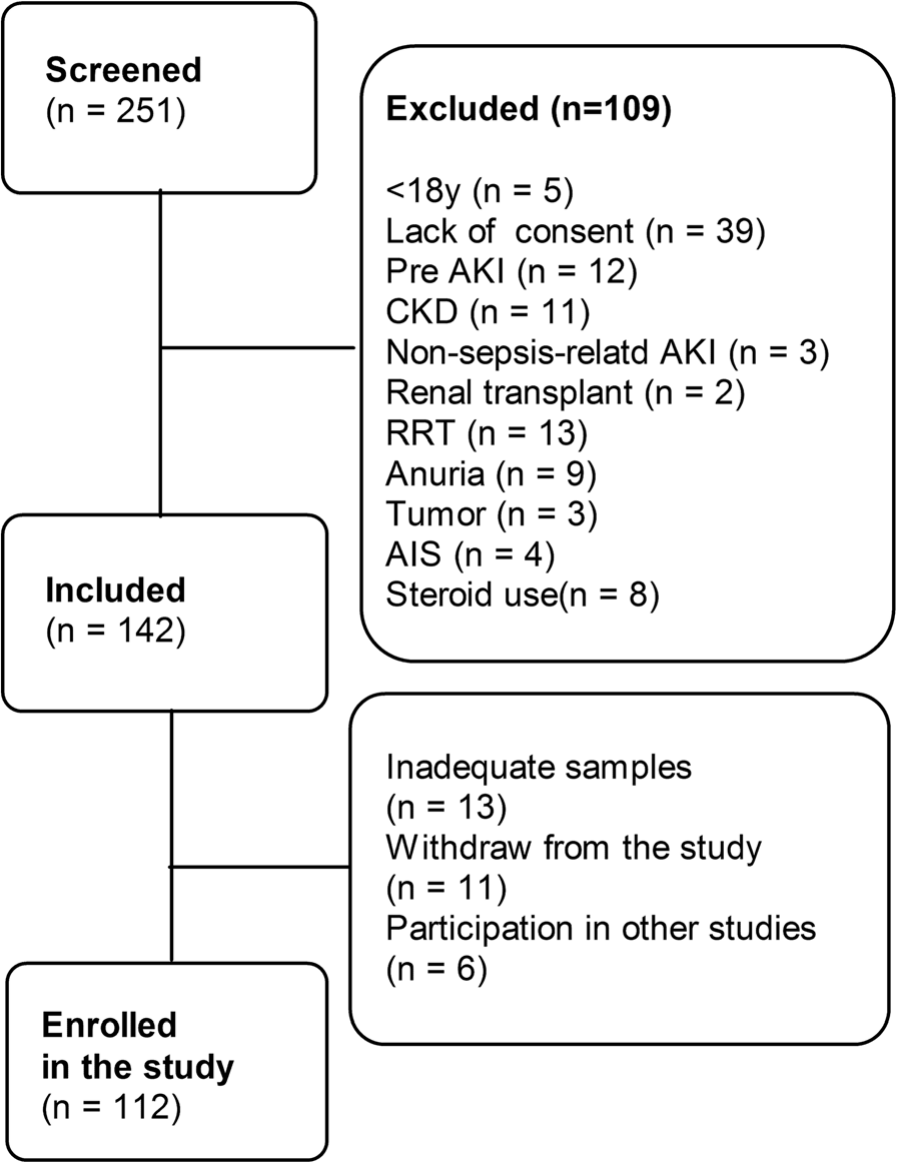
Flow chart depicting the selection process. AIS, acquired immunodeficiency syndrome; AKI, acute kidney injury; CKD, chronic kidney disease; RRT, renal replacement therapy.

**Figure 2:**
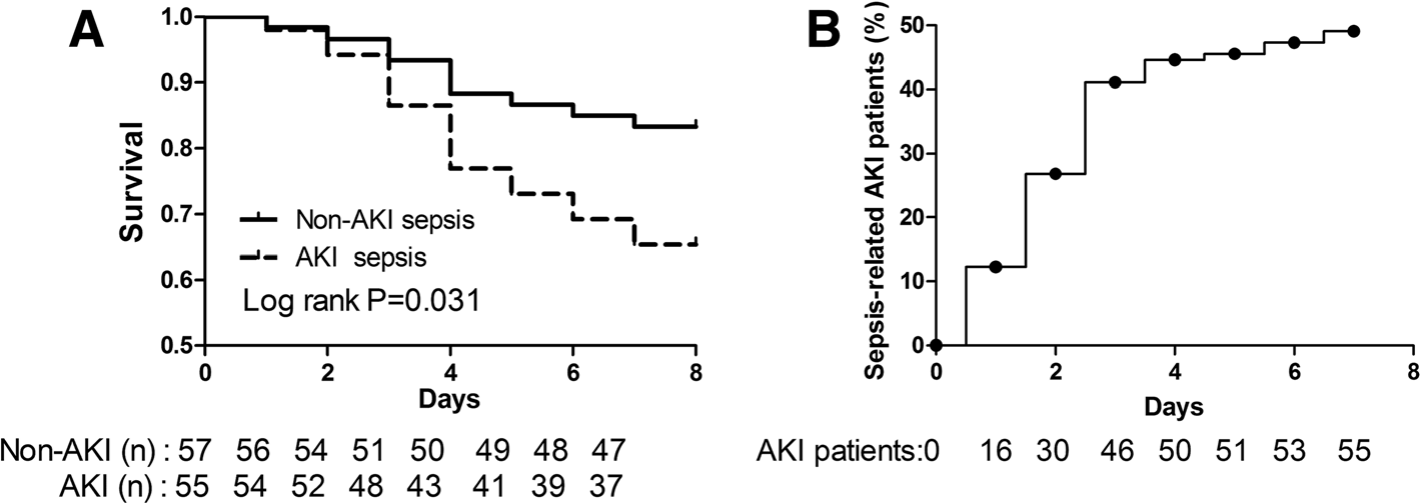
Survival curves and proportion of septic patients who developed acute kidney injury (AKI). **(A)** Survival in sepsis patients with or without AKI. The septic patients who developed AKI trended to a poor prognosis (*P* = 0.031). The log-rank test for trend was used. **(B)** The proportion of septic patients who developed AKI. The number of sepsis-associated AKI patients increased with time during the observation.

In total, 251 consecutive patients were screened. Of these, 109 patients were later excluded according to the exclusion criterion. Of the remaining 142 patients, 30 were excluded for various reasons during the observation period (Figure 1). Finally, 112 were included: 57 without AKI and 55 with AKI. The number of deaths increased with time during the first week in the non-AKI sepsis group and in the AKI sepsis group. Survival analysis showed that the AKI sepsis group had a poor prognosis (hazard ratio = 0.43, 95% CI 0.202 to 0.924, χ^2^ = 4.681, *P* = 0.031) (Figure 2).

Fifty-five patients with sepsis (49.1%) developed AKI based on the KDIGO criteria during the 7-day observation period. The number of sepsis-associated AKI patients increased with time (Figure 2B). The serum PCT level and disease severity scores, including the APACHE II and SOFA scores, were significantly higher in the AKI patients than in the non-AKI patients (*P* <0.01 for all). Both the plasma and urine NGAL, Cys-C, and sTREM-1 levels (*P* <0.01 for all) were significantly different between the two groups. There was no significant difference in age, gender, WBC count, serum CRP, SCr, MAP, mechanical ventilation, etiological factors, or underlying diseases (*P* >0.05 for all) (Table 1).

**Table 1:** **Clinical and biological data on admission**

**Characteristics**	**Non-AKI sepsis**	**AKI sepsis**	***P*** **value**
	**(n = 57)**	**(n = 55)**	
Age, years	51.0 ± 15.6	49.8 ± 15.4	0.664
**Gender, n (%)**			
Male	35 (61.4)	27 (49.1)	0.190
Female	22 (38.6)	28 (50.9)	
**Etiological factors, n (%)**			
Pulmonary infection	17 (29.8)	16 (29.1)	0.549
Abdominal infection	14 (24.6)	17 (30.1)	0.295
Urinary tract infection	9 (15.8)	9 (16.4)	0.569
Trauma-related infection	8 (14.0)	3 (5.5)	0.113
Bacteremia	3 (5.3)	3 (5.5)	0.635
Catheter-related infections	3 (5.3)	3 (5.5)	0.635
Others	3 (5.3)	2 (5.5)	0.525
**Underlying diseases, n (%)**			
Hypertension	35 (61.4)	31 (56.4)	0.406
Diabetes	30 (52.6)	24 (43.6)	0.170
COPD	16 (28.1)	16 (28.1)	0.535
Coronary heart disease	16 (28.1)	15 (27.3)	0.547
Nervous system disease	17 (29.8)	11 (20.0)	0.382
WBC count, ×10^9^/L	15.0 (12.5, 17.8)	16.0 (12.0, 21.0)	0.610
MAP, mm Hg	70.4 ± 14.4	65.9 ± 12.7	0.085
Serum CRP, mg/dL	54.2 (32.2, 60.5)	54.0 (43.3, 67.8)	0.131
Serum PCT, ng/mL	7.0 (3.2, 23.4)	22.1 (6.5, 56.0)	0.002
SCr, μmol/L	79.5 ± 27.9	77.4 ± 33.3	0.721
APACHE II score	15.1 ± 3.4	17.2 ± 4.3	0.004
SOFA score	8.0 ± 2.1	9.4 ± 2.9	0.005
Mechanical ventilation, n (%)	22 (36.7)	28 (53.8)	0.131
Plasma NGAL, ng/mL	123.0 (98.0, 170.5)	165.0 (124.0, 343.0)	<0.001
Urine NGAL, ng/mL	54.0 (43.1, 65.0)	187.0 (76.0, 265.8)	<0.001
Plasma Cys-C, mg/L	1.09 (0.98, 1.22)	1.23 (1.08, 1.45)	0.001
Urine Cys-C, mg/L	0.12 (0.09, 0.13)	0.13 (0.10, 0.32)	0.002
Plasma sTREM-1, pg/mL	98.0 (86.0, 122.15)	132.0 (98.7, 155.0)	<0.001
Urine sTREM-1, pg/mL	43.0 (23.7, 54.9)	54.0 (34.65, 65.0)	0.012

Quantitative data with normal distribution are presented as mean ± standard deviation. Quantitative data with non-normal distribution are presented as median (25th and 75th percentiles). Qualitative data are presented as number (percentage). AKI, acute kidney injury; APACHE II, Acute Physiology and Chronic Health Evaluation II; COPD, chronic obstructive pulmonary disease; CRP, C-reactive protein; Cys-C, cystatin C; MAP, mean arterial pressure; NGAL, neutrophil gelatinase-associated lipocalin; PCT, procalcitonin; SCr, serum creatinine; SOFA, Sequential Organ Failure Assessment; sTREM-1, soluble triggering receptor expressed on myeloid cells-1; WBC, white blood cell.

### Risk for acute kidney injury occurrence in patients with sepsis

On the basis of the results of the univariate analysis, four variables (*P* <0.1) were selected for multivariate analysis. The results of multivariate analysis using logistic regression with GEE are shown in Table 2. The biomarkers analyzed showed significant associations with the endpoint (AKI occurrence) in patients with sepsis (*P* <0.001) based on KDIGO criteria. The significance was apparent even after adjustment for possible confounders in model 2 (SCr) and model 3 (MAP, APACHE II scores, SOFA scores, and PCT in addition to SCr).

**Table 2:** **Acute kidney injury risk indicated by these biomarkers in septic patients analyzed by using logistic regression with generalized estimating equations**

**Variable**	**Crude: model 1** ^**a**^		**Adjusted: model 2** ^**b**^		**Adjusted: model 3** ^**c**^	
	**OR (95% CI)**	***P*** **value**	**OR (95% CI)**	***P*** **value**	**OR (95% CI)**	***P*** **value**
Plasma NGAL	1.013 (1.010-1.015)	<0.001	1.012 (1.009-1.014)	<0.001	1.013 (1.010-1.022)	<0.001
Urine NGAL	1.030 (1.014-1.047)	<0.001	1.029 (1.014-1.045)	<0.001	1.027 (1.011-1.044)	0.001
Plasma Cys-C^d^	1.003 (1.002-1.004)	0.001	1.003 (1.002-1.003)	0.001	1.003 (1.002-1.003)	<0,001
Urine Cys-C^d^	1.010 (1.005-1.014)	<0.001	1.024 (1.018-1.038)	<0.001	1.022 (1.008-1.035)	0.001
Plasma sTREM-1	1.029 (1.021-1.037)	<0.001	1.023(1.016-1.031)	<0.001	1.028(1.018-1.037)	<0.001
Urine sTREM-1	1.038(1.019-1/057	<0.001	1.033(1.016-1.050	<0.001	1.031(1.009-1.053)	0.005

^a^Not adjusted. ^b^Adjusted for serum creatinine (SCr). ^c^Adjusted for mean arterial pressure, Acute Physiology and Chronic Health Evaluation II scores, Sequential Organ Failure Assessment scores, and procalcitonin in addition to SCr. ^d^Odds ratio (ORs) represent the increase in risk per 0.001 mg/L increase in cystatin C (Cys-C). CI, confidence interval; NGAL, neutrophil gelatinase-associated lipocalin; sTREM-1, soluble triggering receptor expressed on myeloid cells-1.

### Dynamic changes in both plasma and urine NGAL, Cys-C, and sTREM-1 levels

**Figure 3:**
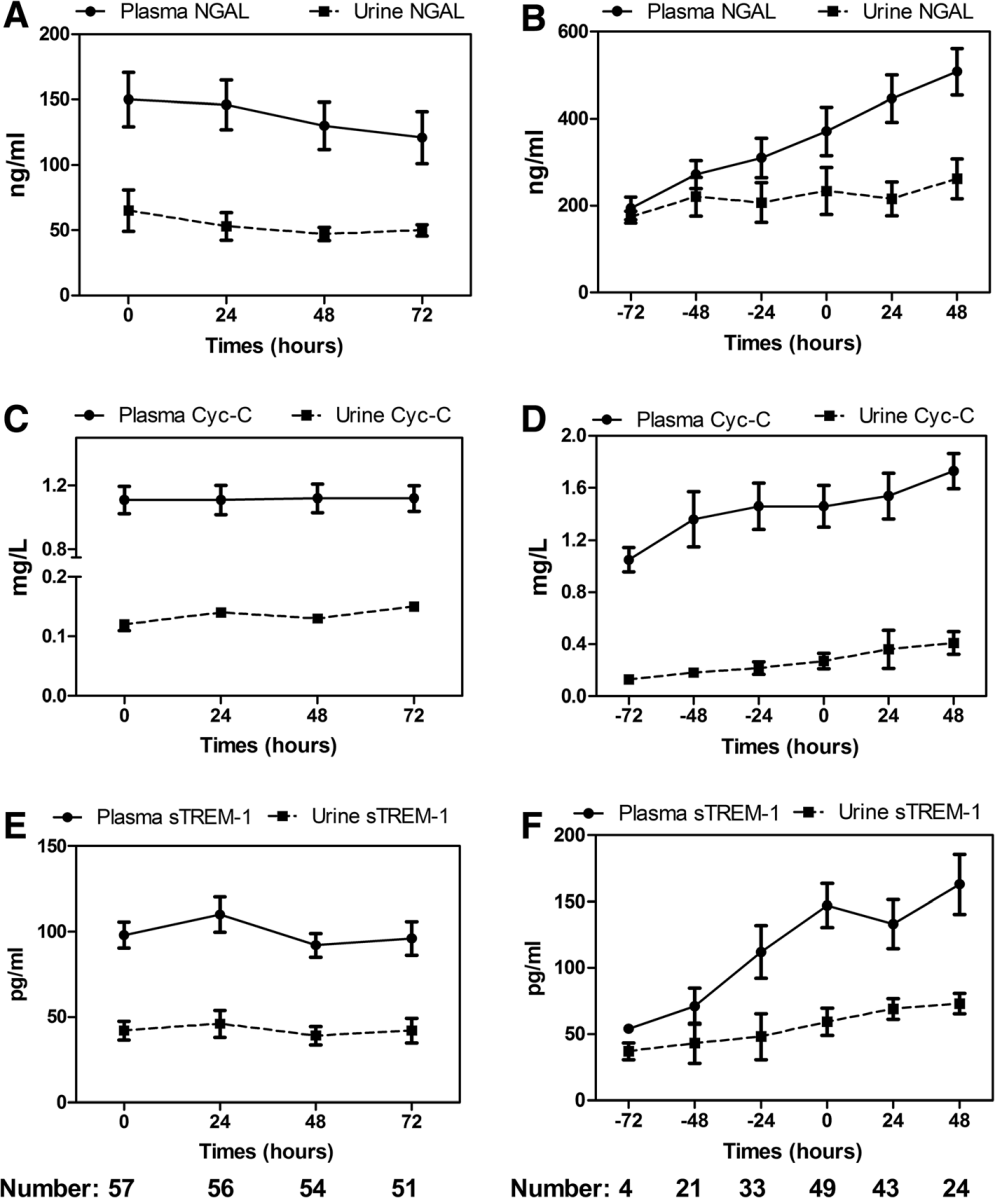
Time course of plasma and urine NGAL, Cys-C, and sTREM-1 levels in non-AKI septic patients **(A, C, E)** and AKI septic patients **(B, D, F)**. For non-AKI septic patients, the time course of these markers in the plasma and urine was explored since their ICU admission. For the AKI septic patients, the time course was from 72 hours prior to AKI development to 48 hours after the endpoint. Data were presented as mean values and 95% confidence intervals. AKI, acute kidney injury; Cys-C, cystatin C; NGAL, neutrophil gelatinase-associated lipocalin; sTREM-1, soluble triggering receptor expressed on myeloid cells-1.

The differences in the levels of these biomarkers at the four different time points at which they were measured (on admission and 24, 48, and 72 hours after admission) were significant (*P* <0.01 for all), and the sepsis-related AKI patients had all-time higher values. In addition, dynamic changes in both plasma and urine NGAL, Cys-C, and sTREM-1 levels on admission and 24, 48, and 72 hours after admission in the septic patients without AKI are summarized and shown in Figure 3. Similarly, the time courses of these markers within 72 hours prior to the endpoint (AKI occurrence) and 48 hours after AKI occurrence in AKI septic patients are shown in Figure 3.

### Diagnostic value of the NGAL, Cys-C, and sTREM-1 levels for AKI occurrence in patients with sepsis

**Figure 4:**
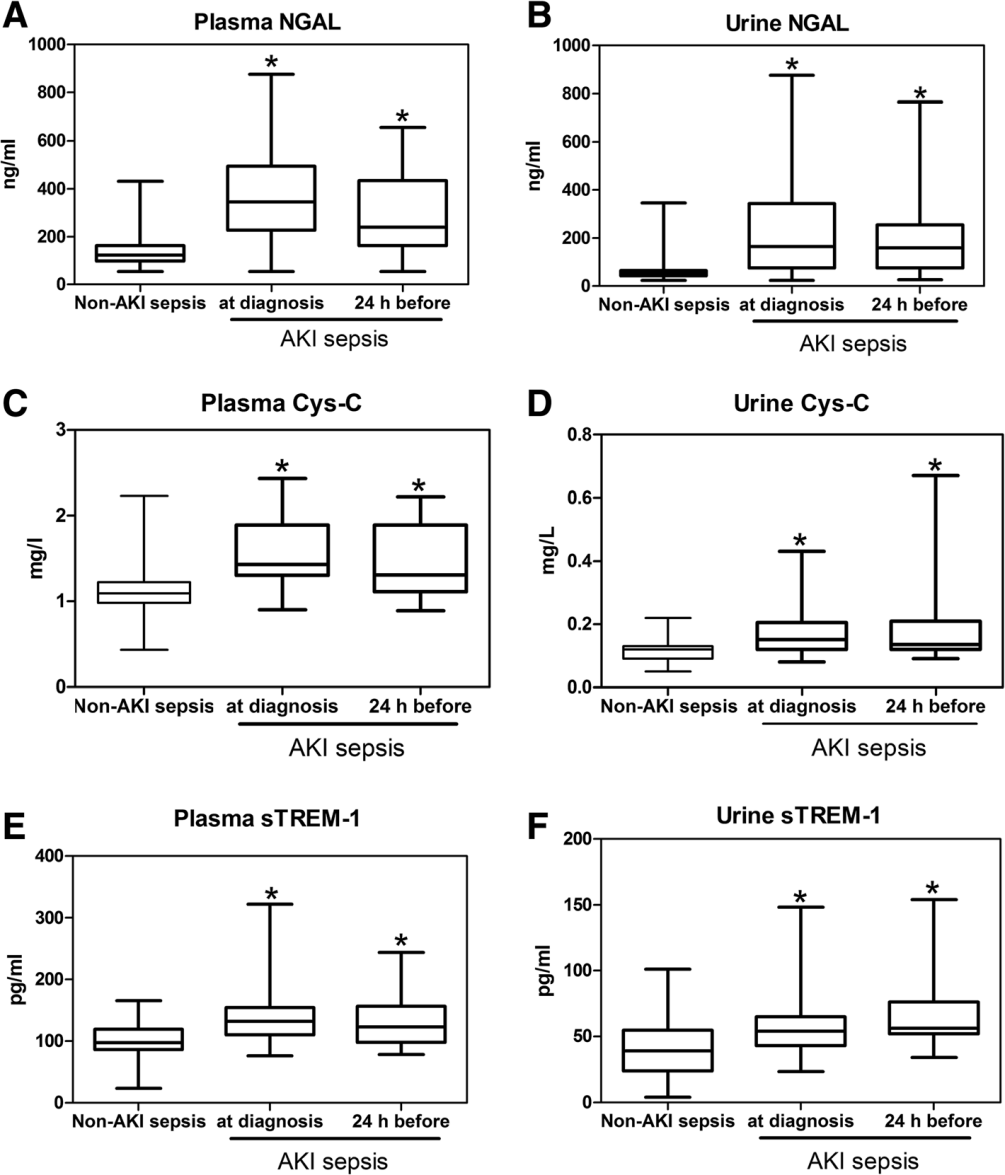
Comparison of the plasma NGAL **(A),** urine NGAL **(B),** serum Cys-C **(C),** urine Cys-C **(D),** plasma sTREM-1 **(E),** and urine sTREM-1 **(F)** levels at the time of AKI diagnosis and 24 hours before AKI diagnosis between the AKI sepsis group and the non-AKI sepsis group. Sepsis-related AKI patients exhibit higher levels of these markers at diagnosis and 24 hours before AKI diagnosis. *P <0.01, compared with the non-AKI sepsis group. AKI, acute kidney injury; Cys-C, cystatin C; NGAL, neutrophil gelatinase-associated lipocalin; sTREM-1, soluble triggering receptor expressed on myeloid cells-1.

**Figure 5:**
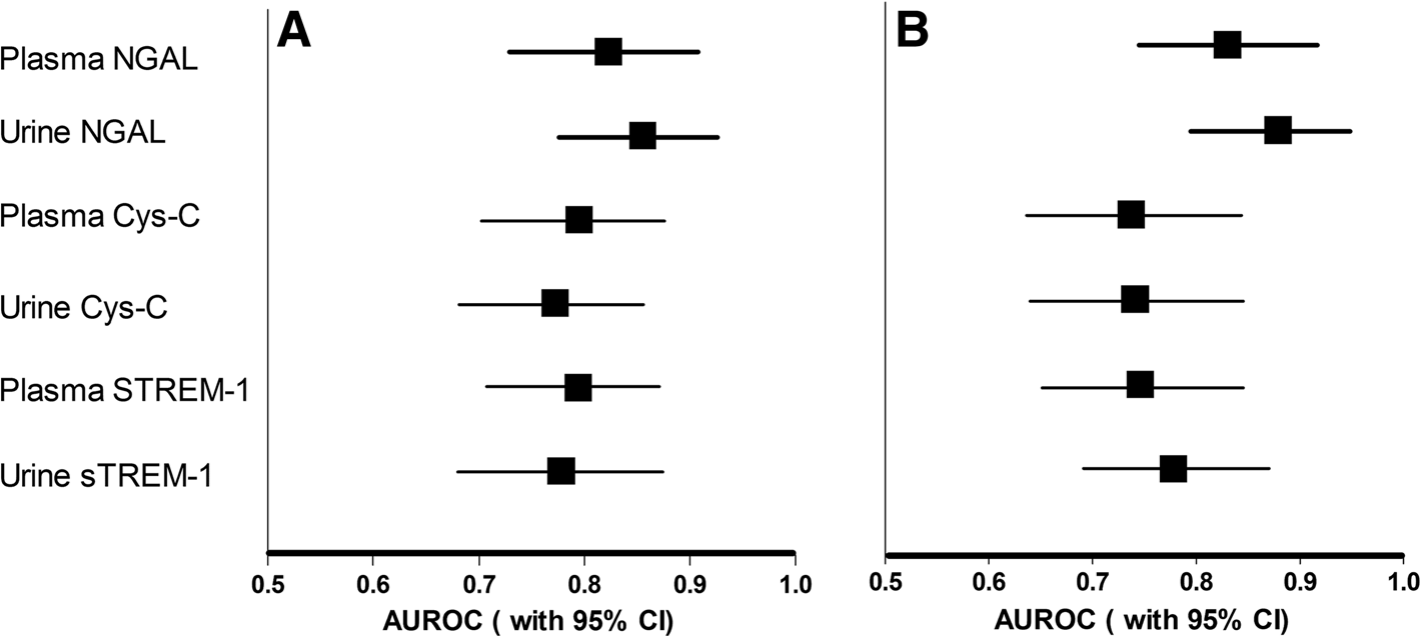
Diagnostic and predictive value of the NGAL Cys-C, and sTREM-1 levels for AKI at diagnosis **(A)** and 24 hours before diagnosis **(B)** in patients with sepsis. Both plasma and urine NGAL performed well for diagnosing and predicting AKI in patients with sepsis, and both plasma and urine Cys-C and sTREM-1 performed fairly well. Data are presented as AUROCs (95% CIs). AKI, acute kidney injure; AUROC, area under the receiver operating characteristic curve; CI, confidence interval; Cys-C, cystatin C; NGAL, neutrophil gelatinase-associated lipocalin; sTREM-1, soluble triggering receptor expressed on myeloid cells-1.

To examine the diagnostic values of plasma and urine NGAL, Cys-C, and sTREM-1 for sepsis-associated AKI, we used the data of 57 non-AKI sepsis patients on admission and 49 AKI sepsis patients at the same time point if AKI diagnosis was based on the SCr values at 9 a.m. or at the next time point if it was based on either urine output or the SCr values at 9 p.m. The data for six AKI sepsis patients were not used since five patients developed AKI after 96 hours and one patient died after AKI diagnosis but before the next test. The NGAL (Figure 4A and B), Cys-C (Figure 4C and D), and sTREM-1 (Figure 4E and F) levels in both the plasma and urine samples of AKI sepsis patients were found to be significantly elevated when compared with those of the non-AKI sepsis patients (*P* <0.01 for all). Figure 5 shows the AUROCs and the 95% CIs for NGAL, Cys-C, and sTREM-1 for AKI occurrence in patients with sepsis. The results indicate that plasma and urine NGAL (AUROC 0.823, 95% CI 0.730 to 0.916 and AUROC 0.855, 95% CI 0.777 to 0.933, respectively) performed well for the diagnosis of AKI occurrence and that plasma and urine Cys-C (AUROC 0.795, 95% CI 0.704 to 0.885 and AUROC 0.772, 95% CI 0.682 to 0.862, respectively) and sTREM-1 (AUROC 0.794, 95% CI 0.708 to 0.880 and AUROC 0.707, 95% CI 0.610 to 0.805, respectively) performed fairly well.

### Predictive values of the NGAL, Cys-C, and sTREM-1 levels for AKI occurrence 24 hours before its diagnosis in patients with sepsis

To assess the predictive values of plasma and urine NGAL, Cys-C, and sTREM-1 for AKI occurrence 24 hours before its diagnosis in patients with sepsis, we used the data of 57 non-AKI sepsis cases on admission and 34 AKI sepsis patients at the last time point before AKI occurrence. The data for 21 patients were not used, because 16 of them in the AKI group developed AKI within the first 24 hours and five developed AKI after 96 hours. We found that 24 hours before AKI occurrence, the AKI sepsis patients had significantly increased plasma and urine NGAL (Figure 4A and B), Cys-C (Figure 4C and D), and sTREM-1 (Figure 4E and F) levels (*P* <0.01 for all) compared with the non-AKI sepsis patients. Figure 5 shows the AUROCs and corresponding 95% CIs for NGAL, Cys-C, and sTREM-1 for AKI prediction at 24 hours before AKI diagnosis in the patients with sepsis. The results indicate that plasma and urine NGAL (AUROC 0.830, 95% CI 0.741 to 0.919 and AUROC 0.879, 95% CI 0.793 to 0.948, respectively) were good indicators of AKI occurrence and that plasma and urine Cys-C (AUROC 0.737, 95% CI 0.633 to 0.841 and AUROC 0.741, 95% CI 0.641 to 0.841, respectively) and sTREM-1 (AUROC 0.746, 95% CI 0.646 to 0.846 and AUROC 0.778, 95% CI 0.687 to 0.870, respectively) also performed fairly well.

## Discussion

In this investigation, we have shown that the plasma and urine NGAL, Cys-C, and sTREM-1 can be used for diagnosing and predicting AKI occurrence in patients with sepsis. Sepsis-associated AKI is associated with a longer length of hospital stay and higher morbidity and mortality rates in critically ill patients [3,16]. Timely diagnosis and effective interventions such as fluid resuscitation, early antibiotic initiation, and restricted use of contrast dye and nephrotoxic drugs at the early stage of sepsis may help to significantly improve the clinical course of the disease. The current diagnostic criteria for AKI, including the RIFLE, AKIN, and KDIGO criteria, all rely on an increase in the SCr level. However, there are some problems associated with this marker in clinical practice. For example, baseline SCr levels are not usually available in the clinical setting. In addition, changes in SCr primarily reflect functional changes in filtration capacity, which may not be valid markers of renal injury. Although a number of studies on new biomarkers for AKI occurrence have been published in the last few years, only a few of them were universally accepted and used in clinical practice.

To evaluate the reliability of NGAL, Cys-C, and sTREM-1 levels for early diagnosis of sepsis-associated AKI, the plasma and urine levels were continuously monitored in the present study. We found that both the plasma and urine NGAL levels were good markers for the diagnosis and prediction of AKI occurrence in patients with sepsis. Recent studies have shown that urine NGAL is a useful biomarker for diagnosing AKI in patients with sepsis [13,21]; however, the diagnostic value of plasma NGAL is under debate. On the one hand, an increasing number of studies, including the present one, indicate that plasma NGAL has good diagnostic value for sepsis-associated AKI [17,31]. On the other hand, Aydogdu *et al*. [21] demonstrated that plasma NGAL is a poor predictor of AKI occurrence in septic patients with an AUROC of 0.44, and a study by Martensson *et al*. [13] showed that it was a poor indicator (AUROC of 0.67) of AKI occurrence in the 12 hours following septic shock. These results can be explained by the reabsorption of NGAL largely by efficient megalin-dependent endocytosis [21]. In keeping with this, Matsa *et al*. [18] reported that the diagnostic accuracy of NGAL for AKI may be more precise if patients with pre-existing kidney disease are excluded. Therefore, our findings conclusively show the diagnostic value of plasma NGAL for AKI occurrence in patients with sepsis since we excluded patients with pre-existing kidney disease.

Cys-C has been considered an early predictor of AKI and an independent predictor of mortality [14,21,24]. In the present study, the AUROC results for both plasma and urine Cys-C indicated that it could be useful in discriminating septic patients with AKI from those without AKI at diagnosis and 24 hours before AKI diagnosis. This has been confirmed in a study by Aydogdu *et al*. [21] which demonstrated that plasma and urine Cys-C are good markers for the early diagnosis of sepsis-associated AKI (AUCs of 0.82 and 0.86, respectively, and thresholds of 1.5 and 0.106 mg/L, respectively); however, their study included patients who had cancer as well as those undergoing steroid treatment and RRT. However, a few studies in adults [12,32] as well as neonates [22] showed that sepsis had no impact on the plasma or urine levels of Cys-C. Therefore, the diagnostic value of Cys-C for detecting AKI in patients with sepsis needs to be confirmed by future studies with a larger sample.

TREM-1 has been identified as an important cell surface molecule involved in sepsis. It is actively expressed in response to infections, and upregulation of its expression is accompanied by an increase in the release of its soluble form (sTREM-1) [25]. Su *et al*. [16] first reported that urine sTREM-1 was an excellent predictor of AKI occurrence at 48 hours before diagnosis, based on a cutoff point of 69.04 pg/mL (AUROC of 0.922, 95% CI 0.850 to 0.995), a sensitivity of 0.941, and a specificity of 0.76. However, no data were available 24 hours before onset, because samples were collected every other day. To the best of our knowledge, no study has evaluated the diagnostic and predictive value of plasma sTREM-1 for sepsis-associated AKI. Therefore, the present study is the first to show the diagnostic and predictive value of plasma sTREM-1 for AKI occurrence (AUROCs of 0.794 and 0.746, respectively). We also found that urine sTREM-1 was a fairly good predictor at the time of diagnosis (AUROC of 0.707) and 24 hours before diagnosis (AUROC of 0.778). The diagnostic and predictive value of urine sTREM-1 in our study was lower than that in the study by Su *et al*., probably because of the difference in the study population and the use of a higher cutoff value in their study.

Our study makes an important contribution to the current body of studies on timely detection of AKI in patients with sepsis as it provides more conclusive evidence of the potential of these novel biomarkers for diagnosing AKI in sepsis. It is conceivable that the integration of these ideal markers into clinical practice can provide incremental benefits for targeted treatment, improve prognosis, and reduce hospitalization cost. Recently, G_1_ cell cycle arrest biomarkers (insulin-like growth factor-binding protein-7 and tissue inhibitor of metalloproteinases-2), key molecules implicated in AKI, have been identified and validated in independent multicenter cohorts; it was shown that the two markers in urine are superior to existing biomarkers [33]. It would be interesting to explore these biomarkers in future studies.

Our study has several strengths. (1) As far as we are aware, previous studies reported potential biomarkers for the early diagnosis of sepsis-associated AKI in different study populations and under non-uniform criteria. In the present study, we demonstrated the diagnostic and predictive values of potential biomarkers under standardized conditions and strict exclusion criteria in the general ICU. We ruled out potential factors that could affect the accuracy of the results: for example, patients with pre-existing AKI and CKD and those who underwent renal transplant and were under RRT and high-dose steroid treatment were excluded (Figure 1). Although our study involved other interceptive subjects with other conditions, including those with coronary heart disease [34], diabetes [20], and urinary tract infections [35], the proportion of these patients did not significantly differ between the two groups (Table 1). (2) This is the first study on these biomarkers of AKI in septic populations to exclude patients undergoing RRT. Kiers *et al*. [36] observed that Cys-C (13 kDa) concentrations in six septic shock patients undergoing continuous RRT decreased significantly following initiation of continuous veno-venous hemofiltration within the first 24 hours (*P* = 0.04). In the study by Mayeur *et al*. [37], 3 hours of intermittent hemodialysis decreased the Cys-C concentrations by 30%. This confirmed that the serum Cys-C concentrations are altered in patients who undergo RRT. Our future studies will investigate whether NGAL (25 kDa) and sTREM-1 (27 kDa) in the bloodstream could be attenuated by RRT (hemofiltration or hematodialysis), as reported for Cys-C. (3) Recently, NGAL and sTREM-1 have been suggested as key players in different cancers [38,39], but since patients with cancer were excluded in our study, the findings for AKI are still valid.

There are several limitations to this study. (1) This was a single-center study, and the number of patients analyzed was insufficient. The present findings need to be validated in multicenter ICUs with larger cohorts. (2) Previous studies have suggested that these markers might be associated with the prognosis of sepsis-associated AKI [16,18,21,32]. Our study did not analyze the association between these biomarkers and the prognosis of the disease, since the observation period was only 7 days. (3) In line with most studies [13,15,16], we screened ‘day to day’ for AKI occurrence, but the patients were not under ‘point to point’ observation. In our study, the time point of diagnosis was defined as the first observation time point after AKI occurrence, and the time point before that was 24 hours before the diagnosis. It is possible that a small number of patients with sepsis had an abnormal GFR value that was undetected by current criteria prior to admission or that a time lag was present from admission until the first sampling. (4) There was no non-septic AKI group or non-septic non-AKI group for comparison in the present study. It cannot be denied that sufficient comparison with these control groups can increase the persuasive power of diagnostic properties. (5) We also did not exclude patients with thyroid dysfunction, although there is no definitive evidence that thyroid function affects the diagnostic accuracy of these biomarkers in detecting AKI occurrence in critically ill patients [40].

## Conclusions

Our study shows that the septic patients who develop AKI have continuously elevated NGAL, Cys-C, and sTREM-1 concentrations in both the plasma and urine compared with those without AKI from admission up to 72 hours after admission. Moreover, these biomarkers showed good validity for the diagnosis and prediction of AKI occurrence in patients with sepsis. Owing to the limitations of our observation, multicenter prospective studies are needed to provide further proof for the clinical diagnostic and predictive value of these potential biomarkers.

## Key messages

- Sepsis-associated AKI patients show a continuous increase in both plasma and urine NGAL, Cys-C, and sTREM-1 levels up to 72 hours.
- Sepsis-associated AKI patients exhibit markedly higher levels of plasma and urine NGAL, Cys-C, and sTREM-1 levels at diagnosis and 24 hours before AKI diagnosis.
- Both plasma and urine NGAL, Cys-C, and sTREM-1 showed a significant association with the development of AKI in patients with sepsis, even after adjustment for possible confounders.
- The diagnostic and predictive values of plasma and urine NGAL were good, whereas those of plasma and urine Cys-C and sTREM-1 were fair.

## Abbreviations

AKI: acute kidney injury
AKIN: Acute Kidney Injury Network
APACHE II: Acute Physiology and Chronic Health Evaluation II
AUROC: areas under the receiver operating characteristic curve
CI: confidence interval
CKD: chronic kidney disease
CRP: C-reactive protein
Cys-C: cystatin C
ELISA: enzyme-linked immunosorbent assay
GEE: generalized estimating equation
GFR: glomerular filtration rate
ICU: intensive care unit
KDIGO: Kidney Disease: Improving Global Outcomes
MAP: mean arterial pressure
NGAL: neutrophil gelatinase-associated lipocalin
PCT: procalcitonin
RIFLE: Risk, Injury, Failure, Loss, and End-stage Kidney disease
rpm: revolutions per minute
RRT: renal replacement therapy
SCr: serum creatinine
SOFA: Sequential Organ Failure Assessment
sTREM-1: soluble triggering receptor expressed on myeloid cells-1
TREM: triggering receptor expressed on myeloid cells
WBC: white blood cell
